# Lipase Activation by Poly(Methyl Methacrylate) in Dispersed Solution: Mechanistic Insights by Fluorescence Spectroscopy

**DOI:** 10.1007/s12010-025-05217-0

**Published:** 2025-03-31

**Authors:** André Merz, Jonas Thelen, Jürgen Linders, Christian Mayer, Kerstin Hoffmann-Jacobsen

**Affiliations:** 1 Chemistry Department, Institute for Coatings and Surface Chemistry, Niederrhein University of Applied Sciences, Adlerstr. 32, 47798 Krefeld, Germany; 2 Institute for Physical Chemistry, University of Duisburg-Essen, Universitaetsstr. 5, 45141 Essen, Germany; 3 Present Address: Institute for Pharmaceutical and Medicinal Chemistry, Heinrich Heine University Düsseldorf, 40225 Düsseldorf, Germany

**Keywords:** Lipase, Activation, Fluorescence correlation spectroscopy, Hydrophobic patch, Poly(methyl methacrylate)

## Abstract

We investigated the mechanisms of polymer-lipase interactions that govern the catalytic activity of lipases in the presence of polymers. Using a combination of fluorescence correlation spectroscopy (FCS), activity analysis, fluorescence spectroscopy, and computational surface analysis, three model lipases—*Thermomyces lanuginosus* lipase (TLL), *Candida antarctica* lipase B (CalB), and *Bacillus subtilis* lipase A (BSLA), with different degrees of hydrophobic active site exposure were studied. Low-molecular-weight poly(methyl methacrylate) (PMMA), synthesized via ARGET ATRP, was employed to study the effect of unstructured polymers in dispersed solution on lipase activity. PMMA significantly enhanced TLL and BSLA hydrolytic activity, while no CalB activation was observed. FCS analysis indicated that this activation was facilitated by polymer lipase binding, a phenomenon observed with TLL and BSLA but not with CalB. Computational analysis further revealed that the surface properties of the lipases were critical for the lipases’ susceptibility to activation by PMMA. Although CalB exhibited the largest total hydrophobic surface area, its homogeneous distribution prevented activation, whereas strong, localized hydrophobic interactions allowed PMMA to bind and activate TLL and BSLA. Supported by the quantitative correlation between elevated 8-anilino-1-naphthalenesulfonic acid (ANS) fluorescence in the presence of PMMA and lipase activity, the activation was attributed to locally increased hydrophobicity of the lipases upon polymer binding. These findings provide critical insights into the role of polymer interactions in lipase activation and stabilization, highlighting the potential for designing tailored polymer carriers to optimize enzyme performance in industrial and biotechnological applications.

**Supplementary Information:**

The online version contains supplementary material available at 10.1007/s12010-025-05217-0.

## Introduction

Lipases (triacylglycerol hydrolases E.C. 3.1.1.3) are a part of the hydrolase family and can be found widely in plants, animals, and microorganisms such as bacteria, fungi, and yeast [1]. Their exceptional regio- and enantioselectivity make them extremely valuable in organic synthesis, especially for reactions that are difficult to achieve using conventional organic chemistry methods [2]. In addition to their ability to hydrolyze carboxylic ester bonds in hydrophobic substances, lipases can facilitate esterification, transesterification, and interesterification reactions in biocatalytic synthesis [3, 4]. Today, they are employed as biocatalysts in the production of enantiomerically pure compounds, including active pharmaceutical ingredients and intermediates, while also contributing to the development of sustainable processes under mild conditions with reduced side reactions [5–7].

Enzymes are immobilized on solid supports to enable their reuse under non-natural and harsh reaction conditions in biocatalytic applications, transforming them into heterogeneous biocatalysts [8–10]. Despite the many advantages of enzyme immobilization, such as enhanced stability against temperature, pH, and organic solvents, it often results in reduced activity. This decrease in activity is typically attributed to diffusional limitations and reduced conformational flexibility of the enzyme [11, 12]. The broad spectrum of synthetic (co-)polymers provides the opportunity to design the microenvironment of the immobilized enzyme in terms of physicochemical interactions and pore structure [13]. Polymethyl methacrylate (PMMA) is a commonly used carrier material for enzyme immobilization in various fields such as medical devices [9], microfluidics [14], biosensors [15], and biocatalytic synthesis. It is accordingly the major component of the commercial heterogeneous lipase formulations, e.g., Novozyme 435® and CalB immo Plus®.

Controlled polymerization techniques such as ARGET ATRP (Activator Regenerated by Electron Transfer Atom Transfer Radical Polymerization) allow the synthesis of polymers with well-defined molecular weights. ARGET ATRP has also been used to create well-defined polymer brushes on solid surfaces as supports for enzyme immobilization [16]. Immobilizing lipases [17] or a broad variety of enzymes [18, 19] on copolymer brushes of varying chemical composition spanning the hydrophobic-hydrophilic regime revealed enzyme stabilization by enzyme-polymer interaction which depended on the property matching between the surface energy of the enzymes and the polymers. This was rationalized by a stabilizing effect of the interaction of hydrophobic and hydrophilic patches of the protein with the polymer preventing unfolding as well as promoting refolding events.

Most lipases have a lid structure that covers the hydrophobic catalytic center and opens upon interaction with the substrate at the hydrophobic interface thus boosting lipase activity [20–23]. Interfacial enzyme activation via lid opening has been the primary explanation for the positive effects of hydrophobic surfaces on lipase activity as surfactants and polymer surfaces [24–30]. *Thermomyces lanuginosus* lipase (TLL) [31] has a distinct lid structure, which has been demonstrated by mutational studies [32–34] and is further supported by crystal structure [35] and molecular dynamics (MD) analysis [36–38]. *Candida antarctica* lipase B (CalB) [39] can be considered the most efficient and most widely used enzyme in organic synthesis. Despite extensive research, it is so far still elusive whether CalB exhibits a lid. CalB has only a very short lid-like structure (α5 helix) which has been discussed as self-activating [40]. *Bacillus subtilis* lipase A (BSLA) [41] is the smallest lipase known to date. BSLA has no lid, which leaves the active site solvent exposed [42].

However, activating effects upon the interaction with hydrophobic materials have been reported for the lid-deficient lipases CalB [16] and BSLA. Molecular dynamics (MD) simulations [43] have highlighted the role of helices α5 and α10 of CalB in a non-typical form of interfacial activation at hydrophobic surfaces. These helices embed into the hydrophobic regions, thereby ensuring that substrates are bound in a productive orientation near the active site [44, 45]. The activation of BSLA by binding monomeric detergent molecules [46] has been rationalized by the modulation of the enzyme surface upon detergent binding leading to strongly separated hydrophobic and hydrophilic regions [47]. As demonstrated by MD simulation, these Janus particle properties provoke an optimal positioning of the active site at the water-substrate interface, which leads to optimal enzyme–substrate interaction.

The effect of the interaction of different lipases with unstructured PMMA in (dispersed) polymer solutions on enzymatic activity has not been addressed before. In contrast to immobilization on polymer brushes and resin particles, the effects of polymer structure can be excluded. Previously, the activation of a protease by polyelectrolytes in solution has been demonstrated by a combination of fluorescence correlation spectroscopy (FCS) and activity analysis [48]. FCS is a powerful analytical technique to study molecular dynamics and interactions via fluorescence intensity fluctuations within a small focus volume [49–51]. This method provides insights into diffusion coefficients and interactions of fluorescently labeled molecules in real-time at the single-molecule level.

In this study, we examined the mechanisms governing the catalytic activity of lipases in the presence of polymers utilizing a combination of fluorescence correlation spectroscopy (FCS) and activity assays, complemented by fluorescence spectroscopy and enzyme surface analysis. Three lipases with varying exposures of the hydrophobic active site, *Thermomyces lanuginosus* lipase, *Candida antarctica* lipase B, and *Bacillus subtilis* lipase A, served as our model system. By employing low-molecular-weight PMMA synthesized through ARGET ATRP, we investigated the effects of polymers in solution and upon the formation of insoluble aggregates beyond the solubility limit. The interactions between lipases and polymers were analyzed with respect to the surface properties of the lipases, and the activity enhancement resulting from molecular interactions was comprehensively examined.

## Materials and Methods

### Materials

The *Thermomyces lanuginosus* lipase (TLL) was a kind gift from ASA Spezialenzyme GmbH (Wolfenbüttel, Germany). The enzyme was desalted (HiTrap Desalting, Cytiva) before use. Lipase B from *Candida antarctica* was purchased from Strem Chemicals (Newburyport, USA). The gene of the *Bacillus subtilis* lipase A (BSLA WT) in the pET 28(a) vector was kindly provided by Dr. Ulrich Krauß (Forschungszentrum Jülich). The protein was expressed in *E. coli* BL21 (DE3) and purified by nickel affinity chromatography (Ni Sepharose High Performance, GE Healthcare) as described previously [46]. The mutant BSLA W42C was expressed and purified by cation exchange chromatography (HiTrap SP HP cation exchange chromatography column, cytiva) as reported before [52].

Succinic acid, sodiumdihydrogenphosphate, disodiumhydrogenphosphate, glycine, copper(II) bromide, N,N,N′,N″,N″-pentamethyldiethylenetriamine (PMDETA), ethyl α-bromisobutyrate (EBIB), methyl methacrylate (MMA), 8-anilino-1-naphtalensulfonic acid (ANS), 4-methyllubelliferone (4MU), 4-methylumbelliferyl butyrate (4MU-B), and sodium chloride (NaCl) were purchased from Sigma-Aldrich/Merck (Darmstadt, Germany). Acetonitrile, ascorbic acid, anisole, methanol, dimethyl sulfoxide (DMSO), acetic acid, and chloroform were from Carl Roth (Karlsruhe, Germany). Atto 488 NHS-ester and Atto 488 maleimide were purchased from ATTO-TEC GmbH (Siegen, Germany). 2-[Methoxy(polyethyleneoxy)propyl]trimethoxysilane was purchased from abcr (Karlsruhe, Germany).

For all measurements, a broad-range buffer was used with 5.72 mM succinic acid, 17.1 mM NaH_2_PO_4_, and 19.98 mM glycine [53].

### Synthesis of Polymethyl Methacrylate

Before polymerization, MMA was purified using an aluminum oxide column and distilled under a vacuum. PMMA was synthesized using ARGET ATRP. 9.8 ml (92 mmol) MMA, 20.36 mg (0.12 mmol) ascorbic acid, and 65 µl EBIB (0.44 mmol) were dissolved in 9.3 ml anisole. The synthesis was started after degassing with argon by the addition of 6.3 mg (0.03 mmol) CuBr_2_ and 59.1 µl (0.28 mmol) PMDETA under argon atmosphere and performed for 1 h at 50 °C under argon atmosphere [16]. The product was precipitated several times in methanol and dried overnight. The number average molar mass *M*_n_ of the PMMA was determined by ^1^H-NMR end group analysis [54] to 10,300 g/mol (Fig. S1). Gel permeation chromatography (GPC) analysis using a GRAM column (8 × 300 mm, 10 μm particle size) from PSS (Mainz, Germany), dimethyl acetamide containing 0.01 mol% LiBr as a solvent, and PMMA standards in the range of 100–1.2 10^6^ g/mol) yield a mass average molecular weight of *M*_w_ = 13,700 g/mol and a poly-dispersity of 1.15. For further experiments, a 1.5 mg/ml stock solution of PMMA was prepared by dissolving it in DMSO.

### Fluorescence Spectroscopy

Fluorescence intensity was analyzed with a Varian Cary Eclipse fluorescence spectrometer at 20 °C. Fluorescence spectra of lipases were acquired from a 3 µM lipase solution with an excitation wavelength of 295 nm. Enzymatic activity was determined with 50 µM 4MU-B [16, 55] in the presence of PMMA concentrations between 0 and 22.5 µg/ml. The excitation wavelength was 327 nm and the emission wavelength was 449 nm. The concentration of the lipase was adjusted to 500 nM using the absorbance at 280 nm and the lipases extinction coefficients (ε_280_ (CalB) = 41,285; ε_280_ (TLL) = 37,275; ε_280_ (BSLA WT) = 24,410; ε_280_ (BSLA W42C) = 18900). 4MU-B and PMMA were used as DMSO stock solutions. The final DMSO concentration in all experiments was 2%. 4MU was used for calibration. Substrate autohydrolysis was negligible at all pH levels below 9 (Fig. S2-S3). At pH 9.25, time traces were corrected by the respective autohydrolysis. Michaelis–Menten analysis was also performed with 500 nM enzyme.

For hydrophobicity analysis, 500 nM lipase was incubated with 40 µM ANS at 20 °C. When PMMA was present, PMMA and lipase were mixed before ANS was added. The excitation wavelength was 380 nm and ANS fluorescence intensity was analyzed at 475 nm.

### Fluorescence Correlation Spectroscopy

Fluorescence correlation spectroscopy (FCS) was performed using a home-built microscope in the confocal setup [56]. Measurements were performed on silanized coverslips. The coverslips were cleaned and silanized overnight in a 1% solution of 2-[methoxy(polyethyleneoxy)propyl]trimethoxysilane in methanol with 5% acetic acid as described previously [57, 58]. The modified coverslips were stored in methanol at 4 °C and blown dry with oil-free compressed air before use. TLL and CalB were labeled with Atto 488 NHS-ester at pH 8.3 in 20 mM PO_4_/0.1 M NaCl buffer for 1 h. BSLA W42C was labeled in the same buffer at pH 7.4 using Atto 488 maleimide. The residual dye was removed by chromatography (HiTrap Desalting, Cytiva). The samples were diluted to 15 nM labeled protein as determined by the absorbance at 488 nm. Non-labeled enzyme was added to a final lipase concentration of 500 nM. Fluorescence time traces were measured for 2 min. The respective burst diagrams of the TLL were divided into snippets with a length of 10 s in accordance with previous studies [59] using QuickFit3.0 (German Cancer Research Center (DKFZ)) [60] to remove data that resulted from large agglomerates that could not be analyzed by FCS fitting. The remaining ca. 90 autocorrelation curves per PMMA concentration were fitted to Eq. 1 in the free diffusion model [50] to determine the apparent diffusion constant.

1 $${\varvec{G}}\left({\varvec{\tau}}\right)=\frac{1}{{\varvec{N}}}\boldsymbol{ }\bullet {\left(1+\frac{{\varvec{\tau}}}{{{\varvec{\tau}}}_{{\varvec{D}}}}\right)}^{-1}\bullet {\left(1+{{\varvec{\kappa}}}^{2}\frac{{\varvec{\tau}}}{{{\varvec{\tau}}}_{{\varvec{D}}}}\right)}^{-\frac{1}{2}}$$

${\varvec{N}}$ is the average number of fluorophores within the focal volume. ${{\varvec{\tau}}}_{{\varvec{D}}}$ corresponds to the diffusion time of the fluorophore and ${\varvec{\kappa}}$ describes the ratio length to the diameter of the focal volume.

The exact number of autocorrelation curves used is provided in Table S1. Burst diagrams obtained from CalB and BSLA were completely converted to autocorrelation curves and fitted to Eq. 1 using SymPhoTime64 (version 2.4.4874) by Picoquant GmbH (Berlin, Germany). The reported diffusion constants were determined via the arithmetic mean of the diffusion constants from the individual autocorrelation curves. Each measurement was performed at least in quadruplicate.

### Enzyme Surface Analysis

Hydrophobic patches of the lipases were analyzed using the pep-patch program (version v0.2.0) [61]. The pdb structures 1DTE (TLL, closed Lid), 1DT3 (TLL, open Lid), 1TCA (CalB), and 1ISP (BSLA) were used after the “clean” command of Yasara (Version 24.4.10) [62] for surface analysis. Pymol (version 2.5.4, Schrödinger LLC, New York, NY, USA) was used for molecular visualizations.

## Results and Discussion

### Effect of PMMA on the Activity of Lipases

**Fig. 1:**
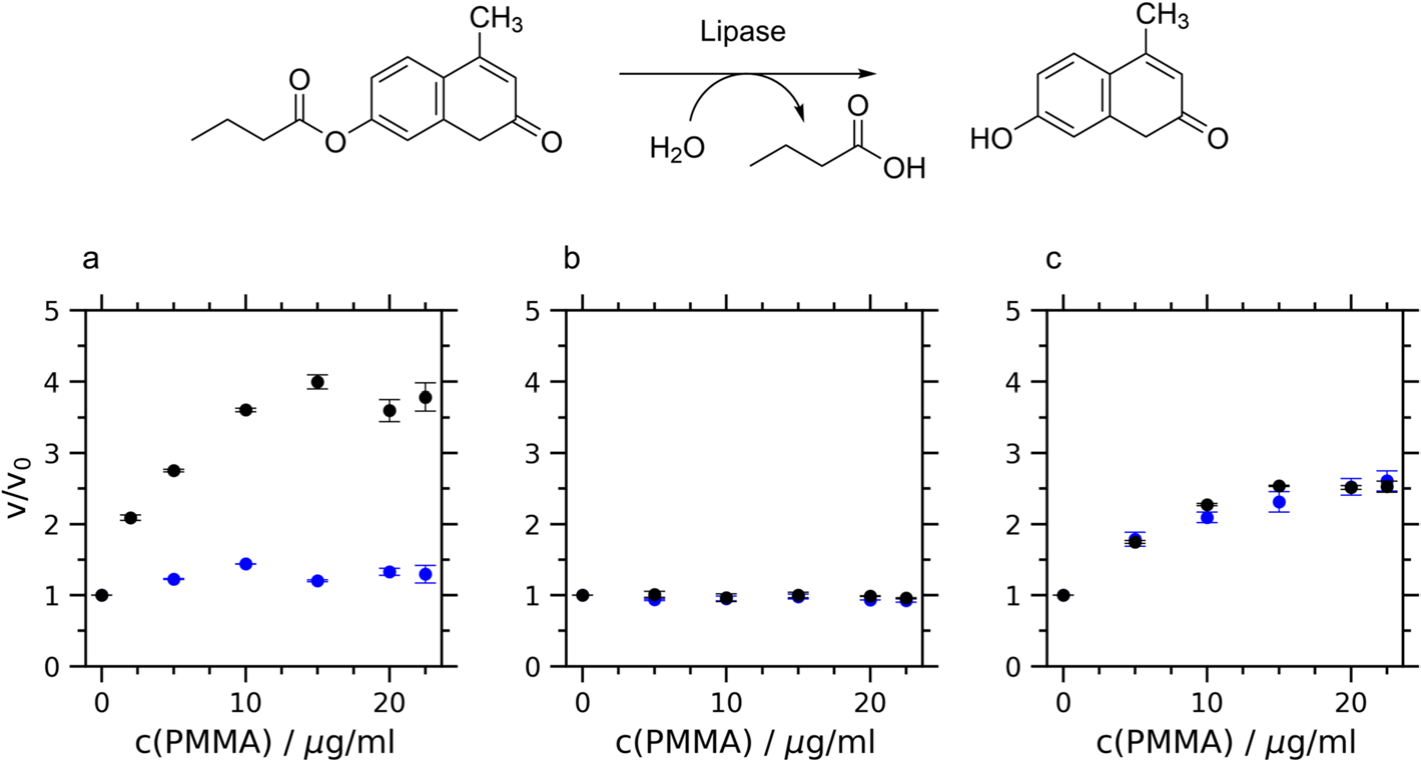
Lipase activity in dependence of the PMMA concentration determined via the relative initial rate of 4MU-B hydrolysis in the presence (*v*) and absence (*v*_0_) of PMMA of **a** TLL, **b** CalB, and **c** BSLA. The analysis was performed at pH 7 (blue) and at the respective isoelectric points (black) of the lipases (TLL, pH 5.00; CalB, pH 5.80; BSLA, pH 9.25)

The hydrolytic activity of the lipases from *Thermomyces lanuginosus* lipase (TLL), lipase A from *Bacillus subtilis* (BSLA), and lipase B from *Candida antarctica* in the presence of PMMA was analyzed using 4-methlyumbelliferone (4-MU) butyrate as fluorogenic substrate (Fig. 1).

As depicted in Fig. 1, the addition of PMMA increased the activities of TLL and BSLA, whereas CalB remained unaffected by the presence of PMMA. Interestingly, the relative activity increase of TLL depended on the pH, showing a strong activity boost at the isoelectric point and only marginal effects at pH 7. The lid-less lipase BSLA showed a more than twofold activity increase with the addition of PMMA at both pH values under investigation.

Enzyme kinetics are commonly described by Michaelis–Menten kinetics with 1/*K*_m_, representing the association constant of enzyme and substrate, and *v*_max_, indicating the reaction rate of this complex. The Michaelis–Menten parameters of the three lipases in the presence and absence of PMMA are summarized in Table 1 and the respective Lineweaver–Burk plots are depicted in Fig. S4-S6. The turnover rates *v*_max_/*K*_m_ (Table 1) supported the findings from the kinetic analysis at a single substrate concentration (Fig. 1), indicating activation of TLL and BSLA by PMMA and a negligible effect of PMMA on CalB activity.

**Table 1:** Michaelis–Menten fit parameters for TLL, CalB, and BSLA WT at pH 7 and their respective isoelectric point in the presence of 0, 5, and 15 µg/ml PMMA

TLL						
	pH 7			pH 5		
c(PMMA)/µg/ml	*v*_max_/µM/min	*K*_m_/µM	*v*_max_/*K*_m_/min^−1^	*v*_max_/µM/min	*K*_m_/µM	*v*_max_/*K*_m_/min^−1^
0	4.16$\pm$0.09	20.8$\pm$1.67	0.2$\pm$0.02	1.41$\pm$0.08	38.3$\pm$5.61	0.04$\pm$0.01
5	6.21$\pm$0.21	37$\pm$3.39	0.17$\pm$0.02	4.64$\pm$0.34	62.9$\pm$11.3	0.07$\pm$0.02
15	12.1$\pm$0.57	78.5$\pm$7.16	0.15$\pm$0.02	9.14$\pm$1.02	86.3$\pm$18.1	0.11$\pm$0.03
CalB						
	pH 7			pH 5.8		
c(PMMA)/µg/ml	*v*_max_/µM/min	*K*_m_/µM	*v*_max_/*K*_m_/min^−1^	*v*_max_/µM/min	*K*_m_/µM	*v*_max_/*K*_m_/min^−1^
0	5.43$\pm$0.12	26.6$\pm$1.77	0.20$\pm$0.02	5.69$\pm$0.13	32.7$\pm$2.20	0.17$\pm$0.02
5	5.64$\pm$0.21	23.4$\pm$2.92	0.24$\pm$0.04	5.10$\pm$0.15	32.9$\pm$2.76	0.16$\pm$0.02
15	4.76$\pm$0.19	22.9$\pm$3.17	0.21$\pm$0.04	4.60$\pm$0.12	23.0$\pm$2.15	0.20$\pm$0.02
BSLA WT						
	pH 7			pH 9.25		
c(PMMA)/µg/ml	*v*_max_/µM/min	*K*_m_/µM	*v*_max_/*K*_m_/min^−1^	*v*_max_/µM/min	*K*_m_/µM	*v*_max_/*K*_m_/min^−1^
0	97.7$\pm$3.4	39.2$\pm$3.61	2.49$\pm$0.32	86.3$\pm$13.2	59.6$\pm$19.8	1.45$\pm$0.70
5	121$\pm$3.61	38.4$\pm$3.07	3.15$\pm$0.35	115$\pm$6.29	43.4$\pm$5.99	2.65$\pm$0.51
15	187$\pm$13.3	38.2$\pm$7.3	4.90$\pm$1.28	177$\pm$12.7	38.9$\pm$7.43	4.55$\pm$1.20

The scattered light by the aqueous PMMA solutions was detected using a fluorescence spectrometer as a semi-quantitative measure of the presence of polymer aggregates in the sample. As depicted in Fig. S7-S8, the light scattering originated from PMMA and not from the substrate, even though the polymer solution appeared clear upon visual inspection. This supported the idea that PMMA was present in dispersed solution.

Michaelis–Menten parameters have to be reconsidered when interfacial processes are involved [63]. *K*_m_ transforms into an apparent value, which is dominated by the adsorption of enzyme and substrate at the interface [25] and is a function of the concentration of the interface-building diluent. As depicted in Table 1, *K*_m_ of TLL kinetics increased ca. threefold in the presence of PMMA. This can be explained by the dilution of the substrate in the interface as the PMMA concentration increases. In the case of interfacial activation, the apparent *v*_max_ represents the enhanced substrate turnover kinetics mediated by the interface for the adsorbed enzyme. *v*_max_ of TLL kinetics increased by a factor of ten in the presence of PMMA.

Enhanced TLL kinetics in the presence of PMMA suggested the presence of interfacial activation of the lipase via lid displacement by the interaction with the polymer in a dispersed solution. This was in line with previous reports of TLL activation by interaction with non-substrate interfaces such as phospholipids [25], which has been attributed to easier substrate access by lid displacement [32, 64]. The role of amphiphilic chemical compounds forming hydrophobic/hydrophilic interfaces has been previously discussed, with TLL activation by surfactant micelles serving as another example [26]. The pH dependency of the activation has been explained by a stronger TLL-micelle affinity at pH 6, resulting from reduced electrostatic repulsion and increased hydrophobic interactions. This leads to the formation of lipase-micelle oligomers, which have been characterized using small-angle X-ray scattering (SAXS) [33, 38, 65].

Activation by lid displacement could be excluded in the case of BSLA. However, *v*_max_ significantly increased in the presence of PMMA whereas CalB showed no activation. Hence, the interaction of PMMA with the different lipases was further analyzed.

### Analysis of PMMA-Lipase Binding by Fluorescence Correlation Spectroscopy

Fluorescence correlation spectroscopy was performed using the fluorescently labeled lipases as spectroscopic probes to analyze attractive interactions between the lipases and PMMA. TLL and CalB were labeled unspecifically via the amine-reactive NHS ester. BSLA was labeled specifically with the cysteine-reactive maleimide dye via the mutant BSLA W42C, as unspecific labeling led to protein aggregation. The kinetic effect of PMMA on BSLA WT and W42C was found to be identical (Fig. S9).

The diffusion constants of CalB and BSLA derived from the experimental autocorrelation curves in the absence of PMMA were in reasonable agreement with the diffusion constants predicted by the Hullrad [33] algorithm, which are 86 µm^2^/s (CalB) and 105 µm^2^/s (BSLA). On the contrary, the diffusion constant of TLL was only half the value predicted for TLL monomer (90.9 µm^2^/s). This revealed that TLL was predominantly present as a dimer under the experimental conditions at both pH, which was in line with previous reports [66].

**Fig. 2:**
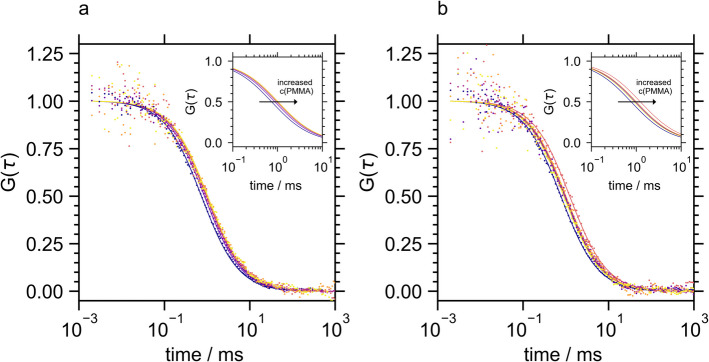
Exemplary fluorescence autocorrelation curves of Atto 488-TLL in the presence of PMMA at pH 7 (**a**) and pH 5 (**b**). Original data is shown as dots and the fits to Eq. 1 as lines using a color code from dark blue to yellow representing increasing PMMA concentrations from 0 to 22.5 µg/ml

The autocorrelation curves of TLL in the presence of increasing PMMA concentration are depicted in Fig. 2. The autocorrelation curves of Atto 488-TLL shifted to higher correlation times with increasing PMMA concentration, indicative of slower diffusion. Using the molecular weight of PMMA of ca. 10,000 g/mol to estimate the radius of hydration (*R*_H_) of PMMA coils by polymer theory of real chains in non-solvent, *R*_H_ ≈ 2 nm was obtained, which is in the order of magnitude of the protein sizes under investigation. Hence, polymer binding should lead to a significant reduction of lipase mobility as observed in Fig. 2.

Analytical analysis of the experimental TLL correlation curves in the presence of PMMA was complicated by the presence of long emission events with a high intensity in the original photon burst diagrams. These events reported the presence of large particles with diameter in the micrometer range, which were assigned to TLL-PMMA agglomerates. If the particle size approaches the scale of the focus dimension, the resulting long bursts cannot be accurately described by an autocorrelation function, leading to distortions in the experimental FCS curve. Figure S10 shows representative burst diagrams, illustrating the increasing occurrence of agglomerates with increasing PMMA concentrations. For analytical analysis of the autocorrelation curve, the burst diagrams were divided into 10 s snippets, and snippets entailing large aggregate bursts were discarded. Hence, the information on the abundance of agglomerate was documented semi-quantitatively in Table S1 deciphering the percentage of evaluable snippets which decreased with PMMA concentration until a PMMA concentration of 15 µg/ml. Accordingly, the remaining FCS curves shown in Fig. 2 accounted only for the dynamics of molecules and particles on the nm-scale which were amenable to FCS analysis. These autocorrelation curves were fitted to Eq. 1 yielding a mean diffusion constant of the complex ensemble (Table 2). Here, a moderate reduction in mobility was observed revealing the formation of lipase-PMMA adducts at both pH levels. It should be noted, that the relative reduction in the diffusion constant upon polymer binding is expected to be less pronounced at lower diffusion constant values. Concluding, the FCS analysis demonstrated an attractive interaction between TLL and PMMA, which led to the formation of single adducts but predominantly larger agglomerates that exceeded the sensitivity range of FCS.

**Table 2:** Mean diffusion constants of the fluorescent-labeled lipases in the presence of PMMA determined from the free diffusion fits to the autocorrelation functions. The errors depict the standard deviation obtained from the analysis of the ca. 90 fluorescence time trace 10-s snippets (TLL) or the analysis of at least a quadruplicate of 2-min time traces (CalB, BSLA)

c(PMMA)/µg/ml	0	5	10	15	20	22.5
**TLL**						
*D*_pH7_/µm^2^/s ± σ	48 $\pm$ 3.5	43 $\pm$ 5.5	40 $\pm$ 4.8	38 $\pm$ 6.38	37 $\pm$ 5.3	39 $\pm$ 4.0
*D*_iso_/µm^2^/s ± σ	48 $\pm$ 3.1	42 $\pm$ 5.4	38 $\pm$ 5.7	36 $\pm$ 8.5	37 $\pm$ 5.5	40 $\pm$ 3.7
**CalB**						
*D*_pH7_/µm^2^/s ± σ	100 $\pm$ 1.9	103 $\pm$ 3.5	92 $\pm$ 5.4	101 $\pm$ 6.8	102 $\pm$ 1.7	101 $\pm$ 4.3
*D*_iso_/µm^2^/s ± σ	88 $\pm$ 3.7	92 $\pm$ 1.4	103 $\pm$ 6.2	85 $\pm$ 4.8	95 $\pm$ 4.9	98 $\pm$ 4.1
**BSLA**						
*D*_pH7_/µm^2^/s ± σ	103 $\pm$ 3.6	93 $\pm$ 2.8	88 $\pm$ 3.9	82 $\pm$ 2.6	92 $\pm$ 3.6	89 $\pm$ 3.2
*D*_iso_/µm^2^/s ± σ	109 $\pm$ 3.9	101 $\pm$ 3.2	103 $\pm$ 1.5	94 $\pm$ 4.5	79 $\pm$ 3.8	75 $\pm$ 2.1

**Fig. 3:**
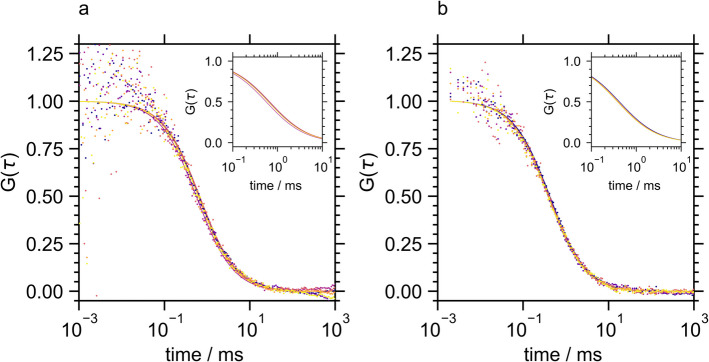
Fluorescence autocorrelation curves of Atto 488-CalB in the presence of PMMA at pH 7 (**a**) and pH 5.8 (**b**). Original data is shown as dots and the fits to Eq. 1 as lines using a color code from dark blue to yellow representing increasing PMMA concentrations from 0 to 22.5 µg/ml

On the contrary, the FCS curve of Atto 488-CalB (Fig. 3, Table 2) and the burst diagrams (Fig. S11) were not altered by the presence of PMMA. This analysis revealed that CalB did not bind to unstructured PMMA in a dispersed solution, indicating a weaker interaction between PMMA and CalB compared to PMMA and TLL. However, the adsorption of CalB on PMMA particles has not only been proven experimentally by hundreds of studies, but PMMA is also the major carrier material for commercial solid CalB formulations. This apparent contradiction suggested that the polymer structure is crucial for the interaction with the lipase. In previous studies, PMMA has been prepared as a polymer brush or porous resin. Here, the importance of brush structure, with intermediate brush densities optimizing immobilized CalB, has been elucidated before indicating that CalB adsorbs in the polymer cavities [16]. We suggest that binding CalB to PMMA requires an interaction between various surfaces of the lipase with the polymer in a three-dimensional form, which can be achieved in porous resins but not with a coiled polymer or unstructured polymer aggregates.

**Fig. 4:**
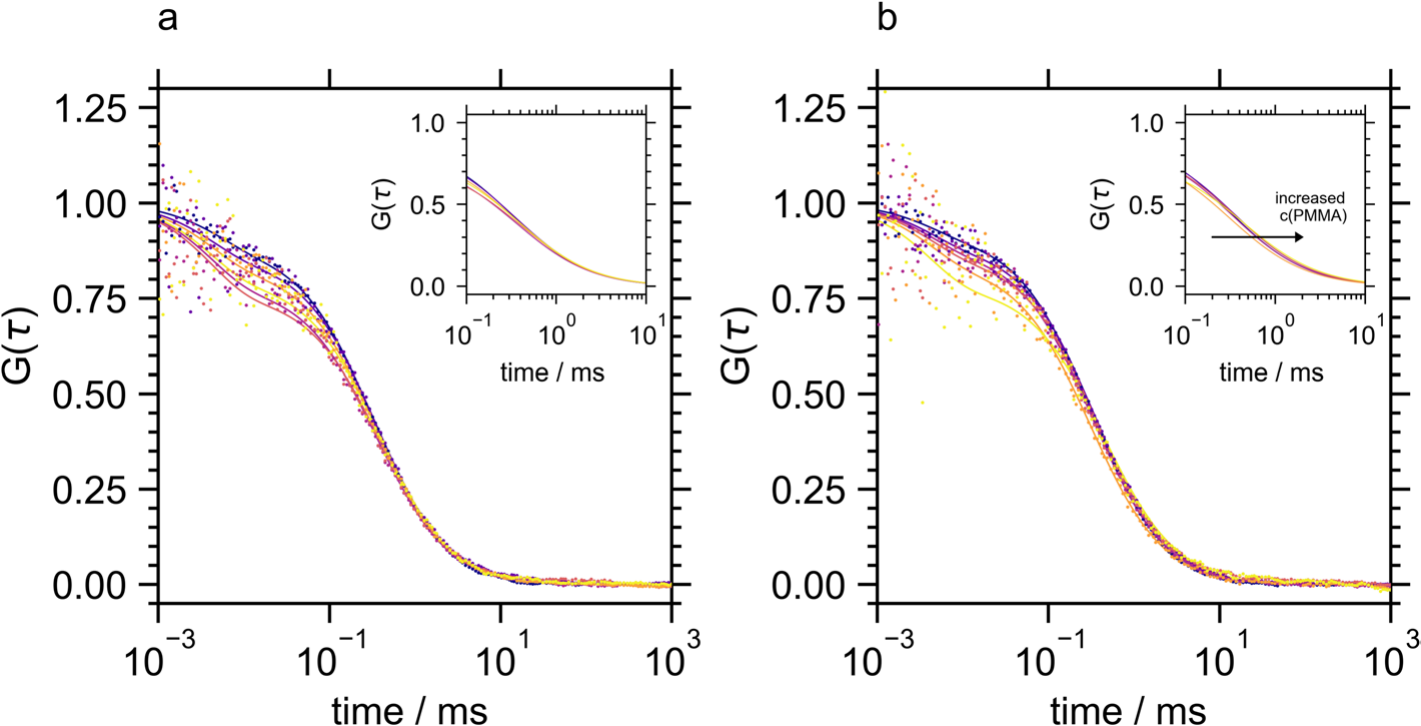
Fluorescence autocorrelation curves of Atto 488-BSLA in the presence of PMMA at pH 7 (**a**) and pH 9.25 (**b**). Original data is shown as dots and the fits to Eq. 1 as lines using a color code from dark blue to yellow representing increasing PMMA concentrations from 0 to 22.5 µg/ml

The fluorescence autocorrelation curves of labeled BSLA were significantly affected by the presence of PMMA. Similar to TLL, the shift to longer correlation times (Fig. 4) indicative of decreasing mean diffusion constants (Table 2) suggested BSLA-PMMA binding. No large aggregates not accessible by FCS analysis were formed (Fig. S12).

To interpret the kinetic activation of the lipases by PMMA, the degree of polymer binding was assessed semi-quantitatively by analyzing the reduction in diffusion constants (Table 2) and, for TLL, by evaluating the number of large aggregates that interfered with the autocorrelation analysis (Table S1) which were then compared with the respective activation (Fig. 1). This analysis revealed that CalB’s resistance to activation by PMMA was due to the absence of binding between CalB and PMMA. In contrast, TLL and BSLA, which could bind to PMMA, were susceptible to activation.

However, the degree of enzyme activation did not align with the semi-quantitative measures of PMMA-enzyme binding determined through FCS analysis. This discrepancy was particularly noticeable in the pH dependencies of binding and activation for TLL and BSLA. FCS analysis demonstrated that PMMA exhibited a strong affinity for TLL, leading to the formation of large enzyme-polymer aggregates at both pH levels. Nevertheless, TLL activation occurred only at its isoelectric point. Conversely, the interaction between BSLA and PMMA displayed a slight pH-dependent binding pattern, yet BSLA activation by PMMA was consistent at both investigated pH values.

This demonstrated that PMMA binding was a necessary prerequisite for lipase activation, suggesting that binding serves as the initial step in the activation mechanism. However, lipase-polymer binding alone was insufficient to fully explain the activation mechanism. The exclusive activation of TLL at its isoelectric point indicated a critical role of hydrophobic interactions in subsequent steps of the activation mechanism, which were further investigated using fluorescence spectroscopy and computational analysis.

### Analysis of Hydrophobic Enzyme Surface Area by ANS Binding

**Fig. 5:**
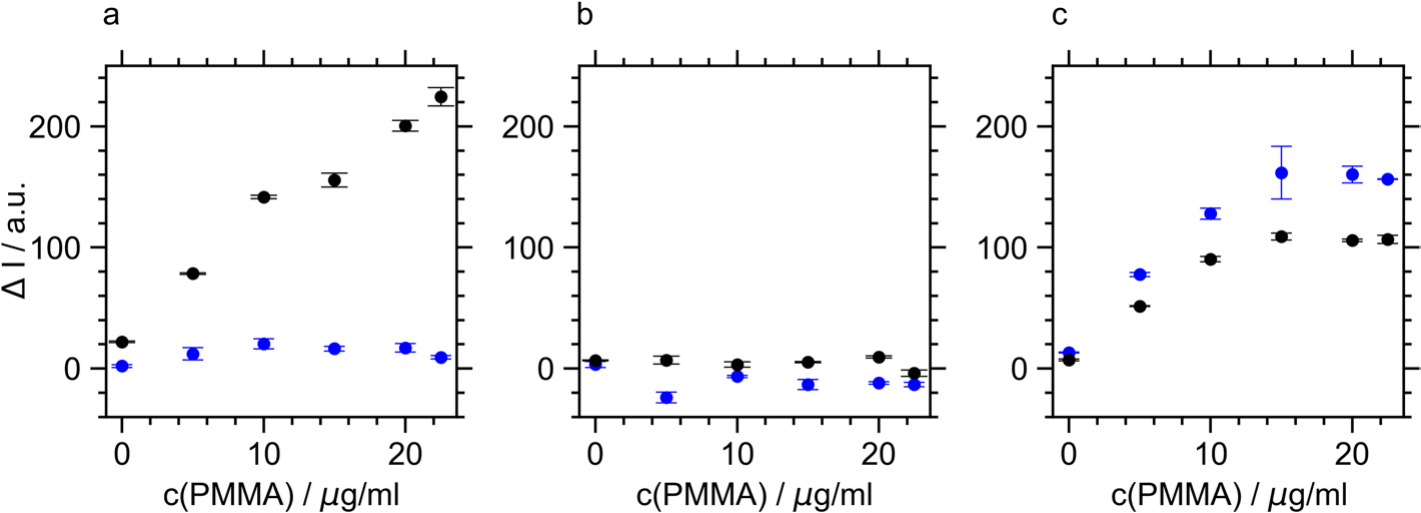
ANS fluorescence intensity in the presence of TLL (**a**), CalB (**b**), and BSLA (**c**) as a function of PMMA concentration at pH 7 (blue) and at the isoelectric points (black) of the enzymes. ANS intensity was corrected with the ANS fluorescence in the presence of the respective PMMA concentration alone

The hydrophobic surface area of the lipases was assessed using the fluorescence of anilinonaphthalene-8-sulfonic acid (ANS), which increases upon binding to hydrophobic (protein) surfaces. Figure 5 presents the ANS fluorescence resulting from hydrophobic surfaces of the lipases in the presence of varying PMMA concentrations.

The addition of PMMA to TLL increased the ANS fluorescence at the lipases’ isoelectric point. This observation aligned with the proposed mechanism of lid displacement due to the interaction of TLL with PMMA, which exposes the hydrophobic substrate cleft and increases the hydrophobic surface area of the lipase. In contrast, PMMA binding did not lead to an increase in ANS fluorescence for TLL at neutral pH. This suggested that TLL activation through lid displacement requires a sufficiently strong hydrophobic interaction between PMMA and TLL, which occurs exclusively at the isoelectric point.

For CalB, the addition of PMMA had no effect on ANS fluorescence, consistent with the absence of lipase-polymer binding observed in the FCS analysis. In contrast, for BSLA, which showed binding to PMMA, ANS fluorescence increased with rising PMMA concentration at both tested pH levels, corresponding to activation occurring at both pH values. These findings emphasized that the interaction of the lipase with PMMA was responsible for the increase in hydrophobic lipase areas. Most importantly, the PMMA-dependent hydrolytic activities of all three lipases showed a statistically significant correlation with their respective ANS fluorescence intensities (Table S2). This correlation indicated that the critical step for lipase activation by PMMA was the increased exposure of hydrophobic surface areas due to the interaction with the polymer. It was particularly intriguing for BSLA, as lid displacement could be ruled out as a contributing factor. Instead, the activation mechanism via increased hydrophobic surface area must arise from conformational dynamics within the lipase, such as changes in the loop region (Tyr129–Arg142), as recently suggested [67]. We propose that the increased hydrophobic area boosts enzyme kinetics by improving substrate binding.

### Analysis of Hydrophobic Protein Surfaces

**Fig. 6:**
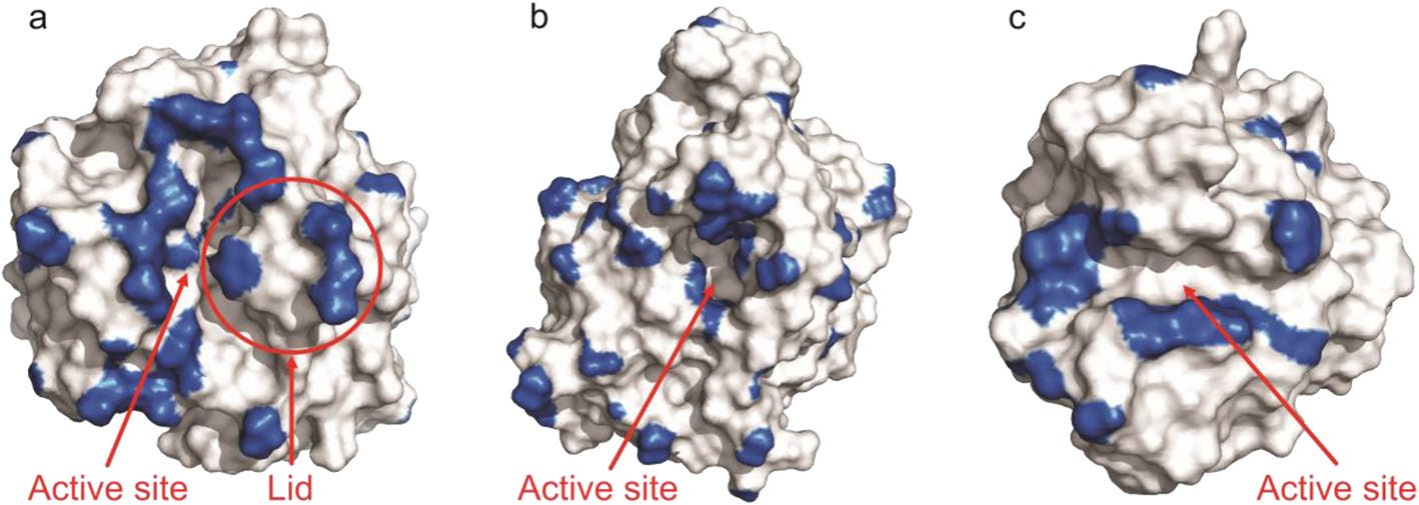
Pep-patch representation of TLL with the lid open (**a**), CalB (**b**), and BSLA WT (**c**) using PyMOL

Recently, methods for quantitative analysis of protein surfaces via the assignment of protein areas with a score for surface forces, i.e., hydrophobicity and electrostatic forces, have been developed [19, 61]. Since the ANS analysis confirmed that hydrophobic interactions with PMMA were responsible for lipase activation, hydrophobic patches were further analyzed using the pep-patch method (Fig. 6). As illustrated in Table 3, CalB exhibits the largest integral hydrophobic surface area alongside the highest number of hydrophobic patches among the three lipases examined. Notably, the distribution of hydrophobic patches on CalB is relatively uniform across its entire protein surface. In contrast, over one-third of the hydrophobic surface area of BSLA is concentrated in and around the substrate cleft, which is readily accessible. The open-lid conformation of TLL reveals the largest contiguous hydrophobic patch, followed by BSLA, TLL in the closed-lid state, and CalB.

**Table 3:** Pep-patch analysis of the hydrophobic surfaces of the lipases TLL [31], CalB [39], and BSLA [41] using the indicated crystal structures from the Protein Data Bank

Enzyme	#Hydrophobic patches	Total surface area/nm^2^	Total area of hydrophobic patches/nm^2^	Area of largest hydrophobic patch/nm^2^
TLL-closed (1DT3)	34	92.8	4.07	1.01
TLL-open (1DTE)	41	97.7	5.34	1.52
CalB (1TCA)	51	102.8	5.94	0.87
BSLA (1ISP)	17	63.9	2.99	1.06

This analysis underscores the significance of the geometric distribution of hydrophobic patches on the lipase surface in influencing the susceptibility of lipases to activation by PMMA. Rather than merely the total hydrophobic surface area, it appears that a nearly unimodal distribution of hydrophobicity along the lipase surface facilitates activation by PMMA in dispersed solution. The observed activation of BSLA by PMMA, in contrast to the lack of activation in CalB, suggests that a lipase’s susceptibility to activation is largely attributable to concentrated hydrophobic interactions within the substrate cleft region.

We suggest that CalB binding to PMMA in a dispersed solution is disfavored by the homogeneous distribution of hydrophobicity on the enzyme’s surface which requires attractive interaction to act from various directions, e.g., in a porous structure. In contrast, the hydrophobic surface area concentrated around the substrate cleft of BSLA enables strong and specific hydrophobic interactions between PMMA and the lipase’s active site region. These interactions increase the hydrophobicity of the active region, thereby promoting activation.

In a recent study investigating the thermal stability of immobilized lipases on polymer surfaces, polymer surface properties were varied via the composition of copolymer brushes comprising different ratios of poly(ethylene glycol) methacrylate (PEGMA) and sulfobetaine methacrylate [19]. The hydrophobic patch analysis of the lipases revealed that the fraction of PEGMA required for optimal lipase stabilization correlated with the free energy of solvation per surface area of the lipase which was determined by the total hydrophobic surface area. Notably, the analysis positioned BSLA and CalB at opposite ends of a hydrophobicity scale, with CalB showing the highest overall hydrophobicity as measured by the free energy of solvation per protein surface area. This contrast emphasizes that lipase stability and activity can both be influenced by the presence of polymers; however, the underlying mechanisms and the impact of polymer properties on enzyme activity and stability can vary significantly. While lipase stabilization is optimized through lipase-polymer interactions at all hydrophobic patches on the protein surface, this study shows that activation occurs through strong local hydrophobic interactions near the substrate cleft.

## Conclusion

This study explored the mechanisms underlying lipase activation by PMMA in dispersed solutions of three structurally distinct lipases, both with and without a lid region, highlighting the role of PMMA binding, hydrophobic surface interactions, and structural requirements for activation. While PMMA binding is the initial step in activation, hyperactivation is driven by increasing the lipases’ hydrophobic surface area via local hydrophobic interactions between PMMA and lipases. Most remarkably, lipase activation by PMMA goes beyond interfacial activation via lid opening as it was found not only for TLL but also for the lid-deficient BSLA. Here, PMMA is suggested to interact with a large hydrophobic patch near the active site, thus expanding the hydrophobic patch in the substrate cleft’s vicinity and enhancing substrate affinity. Interestingly, the surface properties for lipase hyperactivation differed from those found previously for lipase stabilization on polymer brushes. These findings are essential for optimizing lipase immobilization strategies, aiming not only at enzyme stabilization but also at achieving hyperactivation on carrier surfaces. Future research will therefore focus on fine-tuning polymer properties for specific lipase activation.

## Supplementary Information

Below is the link to the electronic supplementary material.

Supplementary file1 (DOCX 328401 KB)

## Data Availability

The datasets generated during and/or analyzed during the current study are available from the corresponding author on reasonable request.
