# Supplementary material for: Lipase Activation by Poly(Methyl Methacrylate) in Dispersed Solution: Mechanistic Insights by Fluorescence Spectroscopy
Source: Appl Biochem Biotechnol. 2025 Mar 31;197(7):4315–31. doi: 10.1007/s12010-025-05217-0 (PMC12316734; doi:10.1007/s12010-025-05217-0)
Supplement: Supplementary file 1 — Supplementary file1 (DOCX 328401 KB) [file 12010_2025_5217_MOESM1_ESM.docx]

# Supplementary Information

Lipase Activation by Poly(Methyl Methacrylate) in Disperse Solution: Mechanistic Insights by Fluorescence Spectroscopy

*André Merz^1,2^, Jonas Thelen^1,✝^, Jürgen Linders^2^, Christian Mayer^2^ and Kerstin Hoffmann-Jacobsen^1^**

^1^ Chemistry Department, Institute for Coatings and Surface Chemistry, Niederrhein University of Applied Sciences, Adlerstr. 32, 47798 Krefeld, Germany

^2^ Institute for Physical Chemistry, University of Duisburg-Essen, Universitaetsstr. 5, 45141 Essen, Germany

Corresponding author:

* [Kerstin.Hoffmann-Jacobsen@hs-niederrhein.de](mailto:Kerstin.Hoffmann-Jacobsen@hs-niederrhein.de)

^✝^ Current address: Institute for Pharmaceutical and Medicinal Chemistry, Heinrich Heine University Düsseldorf, 40225 Düsseldorf, Germany

**Fig. S1** ^1^H NMR (CDCl_3_, 500 MHz) spectrum of poly(methyl) methacrylate synthesized via ARGET ATRP. Signals C and E were used for the calculation of M_n_.

**Fig. S2** Exemplary kinetic time traces of the hydrolysis of 50 µM 4MU-B by 500 nM BSLA WT for the degradation at pH 7 (blue) and pH 9.25 (black) in comparison to the autohydrolysis of the substrate in the absence of enzyme.

Fig. S3 Exemplary kinetic time traces of the enzymatic hydrolysis of 50 µM 4MU-B in comparison to autohydrolysis in the acidic regime. a: In the absence (dotted line) and presence of 500 nM CalB (line) at pH 5.8. b: In the absence (dotted line) and presence of 500 nM TLL (line) at pH 5.

Fig. S4 Lineweaver-Burk plot of 4MU-B hydrolysis by 500 nM TLL in the absence (●) and presence of 5 µg/ml (♦), 15 µg/ml (▲) PMMA at pH 7 (a) and at the isoelectric point pH 5 (b)

Fig. S5 Lineweaver-Burk plot of 4MU-B hydrolysis by 500 nM CalB in the absence (●) and presence of 5 µg/ml (♦), 15µg/ml (▲) PMMA at pH 7 (a) and at the isoelectric point pH 5.8 (b)

Fig. S6 Lineweaver-Burk plot of 4MU-B hydrolysis by 500 nM BSLA WT in the absence (●) and presence of 5 µg/ml (♦), 15 µg/ml (▲) PMMA at pH 7 (a) and at the isoelectric point pH 9.25 (b)

The scattered light from 4MU-B-solutions was determined in the fluorescence spectrometer to exclude an interfacial activation of the lipases by the substrate 4MU-B itself. As depicted in Fig. S7, 4MU-B aggregation occurred well beyond the concentration of 50 µM 4MU-B, which was used for the activity measurements.

Fig. S7 Scattered light measurement with 4MU-B

The formation of PMMA particles was confirmed by the analysis of the scattered light in the fluorescence spectrometer. Although the solution remained clear by visual inspection, the scattered light revealed the presence of a dispersed PMMA solution with increasing particulate content with increasing PMMA concentration (Fig. S8).

Fig. S8 Scattered light measurement of dispersed PMMA in buffer

As depicted in Fig. S9, the mutation W42C for BSLA labeling did not alter the activating effects of PMMA.

Fig. S9 Initial activity v_0_ of 4MU-B hydrolytic cleavage by 500 nM BSLA WT (empty dots) and 500 nM BSLA W42C (filled dots) at the isoelectric point (black) of pH 9.25 for BSLA in the presence of PMMA

The original burst diagrams acquired for FCS analysis are shown in Fig. S10 (TLL), Fig. S11 (CalB) and Fig. S12 (BSLA), demonstrating the presence of large bursts in the diagrams of TLL in the presence of PMMA.

Fig. S10 Fluorescence burst diagrams of 500 nM Atto 488-TLL in the presence of PMMA at pH 7 (a) and pH 5 (b) with a color gradient from dark blue to yellow according to increasing PMMA concentration from 0 to 22.5 µg/ml

Table S1 Analyzable burst diagrams after splitting the 120 s fluorescence burst diagram of Atto 488-TLL into segments of 10 s

|  | pH 7 | pH 5 |
| --- | --- | --- |
| c(PMMA) / µg/ml | %(evaluable curves) | %(evaluable curves) |
| 0 | 100 | 92 |
| 5 | 88 | 73 |
| 10 | 73 | 73 |
| 15 | 31 | 35 |
| 20 | 52 | 50 |
| 22.5 | 58 | 58 |

Fig. S11 Fluorescence burst diagrams of 500 nM Atto 488-CalB in the presence of PMMA at pH 7 (a) and pH 5.8 (b) with a color gradient from dark blue to yellow according to increasing PMMA concentration from 0 to 22.5 µg/ml

Fig. S12 Fluorescence burst diagrams of 500 nM Atto 488-BSLA W42C in the presence of PMMA at pH 7 (a) and pH 9.25 (b) with a color gradient from dark blue to yellow according to increasing PMMA concentration from 0 to 22.5 µg/ml

Table S2 Pearson correlation coefficient *r* and *p* value of the correlation between lipase activity and ANS fluorescence intensity at the respective PMMA concentrations of TLL, BSLA and CalB.

|  | r | p |
| --- | --- | --- |
| (v / v_0_) vs ANS fluorescence | 0.93 | 4.2 10^-17^ |
